# A Plea for Aseptic Vaccine Virus and Aseptic Vaccination, with a Case in Point

**Published:** 1894-02

**Authors:** Rosa Engleman

**Affiliations:** Chicago, Professor of the Diseases of Children in the Post-Graduate Medical School of Chicago; Attending Physician to the United Hebrew Charities Dispensary of Chicago


### A PLEA FOR ASEPTIC VACCINE VIRUS AND ASEPTIC VACCINATION, WITH A CASE IN POINT.

By ROSA ENGLEMAN, A. B., M. D., CHICAGO,

Professor of the Diseases of Children in the Post-Graduate Medical School of Chicago; Attending Physician to the United Hebrew Charities Dispensary of Chicago.

On the continent every effort is made to secure aseptic lymph. Its asepticity cannot be otherwise than probable, until the specific germ or tox-albumen of cowpox shall be isolated, and a laboratory product placed upon the market. However, it is not unreasonable to now require that a vaccination abrasion be treated as is any other abraded surface, viz., aseptically. Since the vitiation of bovine virus by twenty or more demonstrable germs cannot as yet be excluded, added infection from skin and air can, at least, be prevented by the antiseptic preparation, operation and dressing of the arm.

Should an ulcer, erysipelas, lymphangitis or phlegmon appear, treat each as you would surgical complications elsewhere. Granting them to be infectious, it follows that their invasion and effects must be minimized.

In the proceedings of the Pediatric Section of the New York Academy of Medicine, reported in the January, 1894, number of the *Archives of Pediatrics*, Dr. J. Lewis Smith describes a case of fatal sepsis and death following vaccination. Dr. J. H. Fruitnight mentions a case of tetanus in this relation. Home and foreign literature contributes its quota of cases. The editorials in an early summer number of the *British Medical Journal* entitled, "Militant Antivaccinators" and "The Vaccination Craze," unintentionally reveal the same unfortunate state of affairs. Consequently, the relevancy and justice of this opposition cannot be overlooked.

Dr. J. Lewis Smith's case is commented upon as follows: "Inasmuch as the symptoms of sepsis had not developed for a week or more, the inference was that the wound became infected subsequent to vaccination. The case taught the lesson that greater care should be taken to protect the vaccination wound against infection."

Doubtless great and general carelessness prevails in this respect, but the criticism is not far-reaching enough, in that it fails to touch the root of the evil, the contamination of the virus itself. Reference has been already made to the unavoidable microbic pollution of the lymph. A little study, or better still, a visit to a vaccine station, will convince one that there are other and many extrinsic sources of defilement. Here, in America, there is no certainty that a cow is prepared or the virus secured in accordance with the dictum of surgical bacteriology. The vaccine stables are private commercial institutions, subject to, absolutely, no Federal, State or municipal regulation. It is not sufficient that the stock be select and healthy, and the stables hygienic; the most rigid adherence to antiseptic principles in the bovine inoculation, subsequent dressing, and securement and preservation of the lymph, should be demanded as well.

An epidemic, if not pan-endemic, of sore arms imperatively calls for organized effort on the part of the profession, in order to effect improvement in the present vaccine supply. Proper investigation, organization and legislation with reference to this, so important a subject, necessitates time, while its exigency requires immediate attention. The emergency might be dealt with through the agency of accredited representatives of the local medical societies. These agents might instruct owners and managers of vaccine stations as to the requirements of the profession; the refusal or acceptance of these requisites to be met on the one hand by a boycott, and on the other hand by the offer of a premium for purer virus. My earnest consideration of this subject was stimulated by a case of thrombophlebitis, pyemia and death that occurred in my practice two years ago.

A nursling, although marasmic and feeble from prolonged confinement to a ward that had been quarantined for an outbreak of measles, was vaccinated because of a smallpox epidemic in the institution. The operation was antiseptically done but, to my astonishment, almost every member of the large ward developed some vaccinal complication.

A recent visit to the stable from whence the virus was then obtained explains these manifestations.

The arm of this child, however, presented nothing abnormal and the wound over the left deltoid was about healed, when the nurse called my attention to an abscess on the superior antero-internal aspect of the left arm. Other abscesses rapidly and successively appeared in the left axillary, infra-scapular and pectoral regions, and finally another was found over the lower part of the internal saphenous vein. Nodulation and ecchymosis, involving the affected vein and contiguous tissue, occurred in each instance. Nasal and aural hemorrhages and the rapidly progressive prostration simply confirmed the original diagnosis.

At the autopsy, I found a thrombus in the cephalic vein but a short distance from the normally foveolated scar. The relations of the cephalic to the basilic, subscapular, thoracic and axillary veins demonstrates the infection, atrium and course. Its transmission through the heart and lungs into the general circulation needs no further explanation. Though not creditable, the case has taught me many lessons, and I trust will rouse the profession to a realization of the septic condition of the vaccine virus in general use.—*Jour. A. M. A.*

